# Repeated Sprint Training vs. Repeated High-Intensity Technique Training in Adolescent Taekwondo Athletes—A Randomized Controlled Trial

**DOI:** 10.3390/ijerph17124506

**Published:** 2020-06-23

**Authors:** Ibrahim Ouergui, Hamdi Messaoudi, Hamdi Chtourou, Matthias Oliver Wagner, Anissa Bouassida, Ezdine Bouhlel, Emerson Franchini, Florian A. Engel

**Affiliations:** 1 High Institute of Sports and Physical Education of Kef, University of Jendouba, Boulifa University Campus, Kef 7100, Tunisia; ouergui.brahim@yahoo.fr (I.O.); hamdimessaoudihamdi@gmail.com (H.M.); bouassida_anissa@yahoo.fr (A.B.); 2 Institut Supérieur du Sport et de l’Education Physique de Sfax, Université de Sfax, Sfax 3000, Tunisie; h_chtourou@yahoo.fr; 3 Activité Physique, Sport et Santé, UR18JS01, Observatoire National du Sport, Tunis 1003, Tunisie; 4 Department of Sport Science, Bundeswehr University Munich, 85579 Neubiberg, Germany; matthias.wagner@unibw.de; 5 Laboratory of Cardio-Circulatory, Respiratory, Metabolic and Hormonal Adaptations to Muscular Exercise, Faculty of Medicine, Ibn El Jazzar, Sousse 4000, Tunisia; ezdine_sport@yahoo.fr; 6 Martial Arts and Combat Sports Research Group, School of Physical Education and Sport, University of São Paulo, 05508-030 São Paulo, Brazil; emersonfranchini@hotmail.com

**Keywords:** combat sports, training, fitness, performance, physiological responses, RST

## Abstract

This study investigated the effects of 4-weeks repeated sprint (RST) vs. repeated high-intensity-technique training (RTT) on physical performance. Thirty-six adolescent taekwondo athletes (age: 16 ± 1 yrs) were randomly assigned to RST (10 × 35 m sprint, 10 s rest), RTT (10 × 6 s Bandal-tchagui, 10 s rest) and control (control group (CG): no additional training) groups. Additionally, to their regular training, RST and RTT trained 2×/week for 4 weeks. Training load (TL), monotony, and strain were calculated using the rating of perceived exertion scale. The progressive specific taekwondo (PSTT), 20 m multistage shuttle run (SRT_20m_), 5 m shuttle run, agility T-test, taekwondo-specific agility (TSAT) and countermovement jump (CMJ) tests were performed before and after 4 weeks of training. Additionally, taekwondo athletes performed specific taekwondo exercises (i.e., repeated techniques for 10 s and 1 min). From week 1, mean TL increased continuously to week 4 and monotony and strain were higher at weeks 3 and 4 (*p* < 0.001). VO_2max_ calculated from SRT_20m_ and PSTT increased for RST and RTT in comparison to CG (*p* < 0.001). Agility performance during T-test and TSAT (*p* < 0.01) improved in RTT. The number of performed techniques during the 10 s specific exercise increased in RTT and RST (*p* < 0.01) for the dominant leg and in RTT for the non-dominant leg (*p* < 0.01). The number of techniques during the 1 min specific exercise was higher in RST and RTT compared to CG for the dominant leg (*p* < 0.001). Delta lactate at post-training was lower for RTT for both legs compared to RST and CG (*p* < 0.01). It is important to include a low-volume high-intensity training based on repeated sprint running or repeated technique in the training programs of adolescent taekwondo athletes.

## 1. Introduction

Taekwondo is a striking combat sport which is spreading worldwide [1]. A typical taekwondo match consists of three 2 min rounds with 1 min rest in between, contested in an 8 m × 8 m area [2]. Taekwondo is characterized by the use of a range of offensive and defensive techniques executed at high intensity followed by periods with low intensities [3] resulting in effort to rest ratios varying from 1:2 to 1:7 [1].

Various training modalities have been investigated to optimize the physical fitness of athletes aiming to sustain intensive loads and properly respond to the physiological and physical demands of combat sport competitions [4,5,6,7,8]. Due to the nature and demands of combat sport, sprint interval training (SIT), repeated sprint training (RST) and high-intensity interval training (HIIT) were usually prescribed and implemented by taekwondo coaches [4,7,8,9]. HIIT is defined as repeated efforts of short (<45 s) to long (2–4 min) bouts of high but not maximal-intensity exercise. RST is composed by very short all-out actions (i.e., 5–8 s) interspersed by comparatively long rest intervals (i.e., 40–60 s). However, SIT is composed by all-out sprints of long durations (i.e., >20–30 s) interspersed by 3–4 min rest periods [10].

Recent systematic reviews reported that HIIT was effective to improve the aerobic and anaerobic power and capacity of adult combat sports athletes [9,11]. More in detail, Kim et al. [6] reported that 8 weeks of long duration HIIT induced anaerobic power improvements with blood lactate concentration [La] decreases for adult judo athletes. Likewise, Farzad et al. [4] showed that 4 weeks of RST induced significant improvements in maximum oxygen consumption (VO_2max_) and aerobic power among wrestlers. Considering striking combat sports where effort–pause ratios are quite different from the grappling combat sports investigated in the above-cited studies, it may be important to adapt the HIIT protocols to taekwondo-specific loads. 

Recent studies have investigated the effect of HIIT among taekwondo [7,12] and karate [8] athletes and reported aerobic and anaerobic fitness improvements. However, while Monks et al. [7] and Ravier et al. [8] used generic HIIT programs and non-sport-specific tests in pre- and post-tests, Kamandulis et al. [13] used both specific training and sport-specific tests in pre- and post-testing. To improve performance in striking combats sports athletes, training should be specific to the sport demands [13]. For that reason, coaches of different combat sports can use a variety of exercises that ensure the improvement of both the physical and technical–tactical aspects of the athletes [5]. In this consideration, Haddad et al. [5] suggested that specific taekwondo technical interval training, performed with high to maximum intensities, could be an effective training modality for athletes improving both taekwondo-specific techniques and cardiorespiratory capacities. Indeed, the authors reported that repeated taekwondo technique training induced the same intensities as short interval running in adolescent athletes (i.e., similar heart rate and rating of perceived exertion) [5].

Furthermore, a recent meta-analysis [14] and another original research study with taekwondo athletes [12] suggested that HIIT protocols might be feasible in adolescent athletes and were reported to be an efficient strategy to improve aerobic and anaerobic performance when added to sport-specific skill training [12,14]. 

Based on the above-cited studies related to striking combat sports [7,12,13], i.e., those comparing different training methods and in different combat sports, it could be assumed that the results could not be generalized and are specific to each discipline. Moreover, based on our recent literature search, no previous study has compared the RST vs. repeated high-intensity taekwondo-specific technique training on physical and physiological responses in adolescent athletes. 

Thus, the aim of the present study was to compare the effects of 4 weeks of RST vs. repeated high-intensity technique training (RTT) on physical and physiological responses to generic and taekwondo-specific aerobic and anaerobic tests, training load (TL), monotony, strain and perceived recovery (PRS). We hypothesized that the taekwondo-specific high-intensity technique interval training may induce better aerobic and anaerobic adaptations in specific taekwondo tests in comparison to RST.

## 2. Materials and Methods 

### 2.1. Experimental Approach to the Problem 

To compare the different training modalities. Adolescent taekwondo athletes were randomly assigned to an RST, an RTT and a control (CG) group. A simple randomization was established where the random assignments were prepared using excel spreadsheet (Microsoft Office Excel 2007). The interventional period started following two weeks of detraining to eliminate the effects of previous training programs. Additionally, to their usual taekwondo training, the RST and RTT groups performed a total of eight instructed and supervised training sessions (two sessions per week) over a period of 4 weeks. The control group (CG) maintained the habitual taekwondo training routine and participants were asked to refrain from any additional physical activity or training during the study duration.

**Figure 1:**
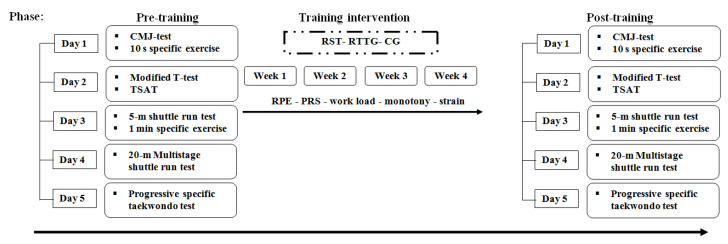
Schematic representation of the study design. CMJ: countermovement jump; TSAT: taekwondo specific agility test; RST: repeated sprint training group; RTTG: repeated technique training group; CG: control group; RPE: rating of perceived exertion; PRS: perceived recovery.

The post-intervention assessment in all groups took place 72 h after the final scheduled training session to warrant sufficient recovery for participants. All tests were conducted at the same time of day to overcome the diurnal variation of performance [15]. Participants were advised to avoid any strenuous exercises in the day preceding all test sessions. All athletes were familiarized with the experimental procedures of all pre- and post-tests in a habituation session. Furthermore, athletes performed the following generic and taekwondo-specific tests: the 20 m multistage shuttle run test [16], the progressive specific taekwondo test (PSTT) [17], the 5 m shuttle run test [18], the modified agility T-test [19], the taekwondo-specific agility test (TSAT) [20] and the countermovement jump (CMJ). Additionally, athletes performed specific taekwondo exercises during which athletes executed the maximum number of technique (i.e., Aptchagui) for 10 s as well as for 1 min, both with the dominant and the non-dominant leg [21]. These exercises were chosen since it has been reported that [La] and heart rate responses were correlated with those induced by a simulated taekwondo combat [21]. All pre- and post-tests were performed during different sessions allowing a sufficient recovery periods (48 h to 72 h) to avoid any fatigue effects between sessions. A sufficient recovery period between tests from the same session (i.e., session 1 to 3 in Figure 1) was given for all athletes (≥ 30 min). The parallel study design is presented in Figure 1.

### 2.2. Participants

**Figure 2:**
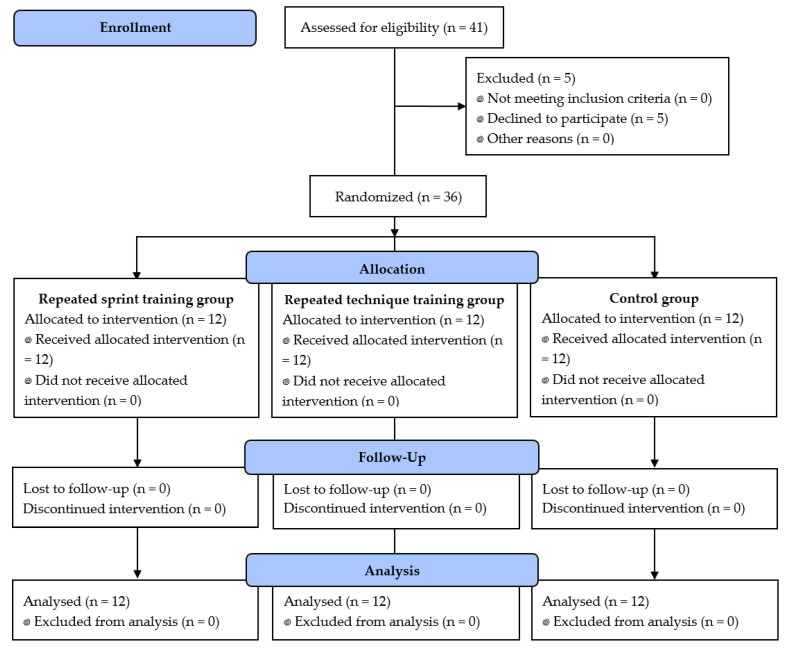
Participants flow.

The required sample size was calculated at posteriori using the software G*Power (version 3.1.9.2; Kiel University, Kiel, Germany). Based on the study of Monks et al. [7], the effect size was estimated to be 0.5 and α and power were set at 0.05 and at 0.80, respectively. Accordingly, the required minimal sample size for the present study was 33 participants. Of 41 athletes recruited, 41 were eligible, 36 agreed to participate, and 12 were randomly assigned to each group. At baseline, there were no imbalances in the characteristics of the three groups. The participants flow is presented in Figure 2. 

Thirty-six taekwondo athletes (27 males and 9 females) competing at regional and national level were randomly assigned to one of three groups: RST (*n* = 12; age: 16 ± 1 years; height: 165.8 ± 7.1 cm; body mass: 56.9 ± 7.2 kg; body mass index (BMI): 20.7 ± 2.3 kg/m^2^), RTT (*n* = 12; age: 16 ± 1 years; height: 167.4 ± 8.3 cm; body mass: 55.6 ± 8.0 kg; BMI: 19.9 ± 2.0 kg/m^2^) and CG (*n* = 12; age: 16 ± 1 years; height: 162.3 ± 9.9 cm; body mass: 56.8 ± 12.7 kg; BMI: 21.4 ± 3.3 kg/m^2^), with female participants were equally assigned to the study groups. Athletes trained regularly for 2–7 years with an average of 2 ± 1 training sessions per week lasting two hours per training session. Athletes had been competing regularly (regional and national tournaments) for 2–7 years. Participants did not present any medical conditions or acute or chronic injuries during the whole experimentation period. The study was conducted according to the Declaration of Helsinki for human experimentation [22] and the protocol was fully approved by the local research ethics committee before the start of the study. All athletes, their parents, and their coaches gave written informed consent after a detailed explanation about the aims, benefits, and potential risks involved in the investigation.

### 2.3. Training Intervention

Athletes performed RST and RTT two times per week for 4 weeks in addition to the regular taekwondo training. The RST consisted of 10 × 35 m sprint running with 10 s of passive rest between repetitions [4]. RTT group completed 10 × 6 s as many repetitions as possible of a taekwondo technique (i.e., Bandal-tchagui), intercepted with 10 s of passive rest between series [4].

For both training groups, the two sessions of the first training week were composed of 3 sets with a rest of 3 min in between. An increase by one set per week was applied (i.e., reaching 6 sets in the last training week [4]). Before each training session, a 15 min warm-up routine was performed, composed by running, rope skipping, stretching, and low-intensity kicks. Additional to RST and RTT training sessions, athletes performed three habitual technical–tactical-based taekwondo training sessions (2 h each) per week.

The CG realized the same habitual taekwondo training sessions as the RST and RTT groups without additional training over the study duration. 

### 2.4. Testing Procedure

Training load and ratings of perceived recovery monitoring.

Fifteen to30 min after each training session, session RPE scores of all athletes were registered, by answering the question “how difficult was the training session?”, to calculate the TL, monotony and strain [23]. Monotony reflects the variability of TL and was calculated by dividing the mean daily load over each week by its standard deviation [24]. Strain represents the general stress caused by the TL and low variability of the practice sessions and was calculated by multiplying monotony by the accumulated weekly training load [24]. Moreover, PRS scores [25] were collected before every training session to monitor recovery status of athletes. Throughout the study, the duration (i.e., including the warm-up, cool-down and rest between the different tasks) and the intensity of each training session were recorded for each athlete. 

### 2.5. The 20 m Multistage Shuttle Run Test

The 20 m multistage shuttle run test [16] consisted of running with continuously increasing velocity back and forth between two lines separated by 20 m until voluntary exhaustion. Athletes started with an initial speed of 8 km/h, which increased by 0.5 km/h every minute. However, the required running velocity in each sequence was controlled by a pre-recorded acoustic signal. The Leger prediction equation was used for the indirect calculation of maximum oxygen uptake (VO_2max_) [16]. The intra-class correlation coefficient (ICC) for test–retest trial from the present study was 0.822.

### 2.6. Progressive Specific Taekwondo Test (PSTT)

The PSTT is a progressive step test until voluntary exhaustion to assess aerobic power and capacity in taekwondo athletes during a taekwondo-specific effort. The PSTT was performed in a 2 m × 2 m pitch, using a punching bag of 1.0 m × 0.9 m. In the first stage participants began with the right leg and performed 6 kicks for 100 s with alternating legs. In the subsequent stages, the number of kicks increases by 4 kicks per stage at the same time the duration of the stages is decreasing. During the test, athletes remained in step position (fighting stance hopping). The kicking frequency was dictated by acoustic signals with fixed intervals between each kick for each stage [26]. During the PSTT, heart rate (HR) was recorded continuously at 0.5 s intervals using HR monitors (Polar Team2 Pro System; Polar Electro, Kempele, Finland) and mean HR (HR_mean_) was calculated. VO_2max_ was indirectly calculated based on the prediction equation determined by Rocha et al. [27]. ICC for test–retest trial from the present study was 0.825.

### 2.7. The 5 m Shuttle Run Test

The 5 m shuttle run test consisted of running as far as possible within 30 s by going back and forth between the start line and lines in the distance of 5 m, 10 m, 15 m, and 20 m. The 5 m shuttle run test is performed 6 times for 30 s with 35 s rest between each trial. During the 35 s recovery period, the athletes returned to the starting position for each of the six repetitions [18]. The covered distance was calculated for every repetition in the nearest one meter. The total distance and the best distance recorded during the test were used for final analysis. Additionally, the fatigue index (i.e., (maximum of distance–minimum of distance)/maximum of distance) [18] was calculated. Blood samples were collected at 10 min before and immediately after the 5 m shuttle run exercise from the fingertip and [La] was measured using the Lactate Pro 2 Analyzer (Arkray, Tokyo, Japan) and only delta change (Δ) values were used for the analysis. ICC for test–retest trial from the present study of the total covered distance was 0.752.

### 2.8. Modified Agility T-test

The athlete started the test with both feet behind the start line. At his/her discretion, the athlete sprinted to the cone ahead of him/her and touched it. Following his/her choice, the athlete shuffled to the left (or right) cone without crossing feet and touched it, then shuffled to the other cone and touched it and shuffled to the cone in front again and finally the athlete ran backward to the start line [19]. The time from start to finish was measured using two sets of single beam timing lights (Brower Timing Systems, Salt Lake City, UT, USA). Three trials were performed by each athlete and the best performance was registered for the analysis, and 2 min rest intervals were allowed between trials. ICC for test–retest trial from the present study was 0.972.

### 2.9. Taekwondo-Specific Agility Test (TSAT)

The athlete began the test with his/her fighting position stance behind the start line. At his/her discretion, the athlete advanced to the center mark as fast as possible, following his/her own choice, he/she turned towards partner 1 by a shift and performed a roundhouse kick with his/her lead leg, then he/she turned to the other side and shifted to partner 2 and performed another roundhouse kick with the other lead leg, after that he/she returned to the center, the athlete moved to partner 3 in guard position and performed a double roundhouse kick, finally the athlete ran backward to the start/finish line [20]. The performance time was measured with two sets of single beam timing lights (Brower Timing Systems, Salt Lake City, UT, USA). Three trials were performed by each athlete and the best performance was documented for the analysis and two minutes of passive rest were allowed between trials. ICC for test–retest trial from the present study was 0.858.

### 2.10. Countermovement Jump (CMJ) 

Athletes started from an upright standing position, completed a fast downward movement by flexing the knees and hips, immediately followed by a rapid extension of both legs. During the extension of the legs a vertical arm-swing of both arms was performed to yield a better performance in the CMJ. The performance was recorded as the height of the jump using an Optojump (Optojump, Microgate, Bolzano, Italy). Three trials were performed, and the best performance was maintained for the analysis. 45 s of passive recovery was allowed between trials. ICC for test–retest trial from the present study was 0.972.

### 2.11. Specific Exercises 

In this exercise test athletes had to perform the direct kick (i.e., Apchagui) as fast as possible applied on a taekwondo racket with the dominant and non-dominant leg for 10 s and 1 min. The total number of kicks executed with the dominant and non-dominant leg during the 10 s and 1 min trial was documented for final analysis. Moreover, [La] was measured before and after the 1 min repeated technique tests using the Lactate Pro^2^ Analyzer (Arkray, Tokyo, Japan) and Δ values were used for the final analysis. ICC for test–retest trial from the present study were 0.86 and 0.87 for dominant and non-dominant legs for the 10 s exercise and 0.87 and 0.80 for the dominant and non-dominant leg during the 1 min exercise, respectively.

### 2.12. Statistical Analyses

Data were presented as mean and standard deviation (SD). The statistical analysis was performed using SPSS 20.0 statistical software (IBM corps., Armonk, NY, USA). The normality of data sets was checked and confirmed using the Kolmogorov–Smirnov test. Sphericity was tested and confirmed using the Mauchly test. Data were analyzed using a two-way analysis of variance (group (RST, RTT and CG) × training (pre and post)) with repeated measurements to compare aerobic and anaerobic performance. Bonferroni was used as post hoc test. Standardized effect size (Cohen’s d) analysis was used to interpret the magnitude of differences between variables and classified according to Hopkins [28]: ≤0.20 (trivial); ≤0.60 (small); ≤1.20 (moderate); ≤2.0 (large); ≤4.0 (very large); >4.0 (extremely large). Moreover, upper and lower 95% confidence intervals of the difference (95% CI_d_s) were calculated for corresponding variation. The statistical significance level was set at *p* ≤ 0.05.

## 3. Results

Weekly TL, monotony and strain values are presented in Table 1. For the weekly TL, there was only a significant training effect (F_3,88_ = 8.3; *p* < 0.001) with values for week 1 being higher than weeks 2 (95% CI_d_ = 11; 112; d = −1.09 (moderate); *p* = 0.009), 3 (95% CI_d_ = 17; 118; d = −1.1 (moderate); *p* = 0.003) and 4 (95% CI_d_ = 38; 139; d = −0.2 (small); *p* < 0.001). 

For monotony, there was only a significant training effect (F_3,88_ = 22.5; *p* < 0.001) with lower values for week 1 than weeks 3 (95% CI_d_ = −6;−3; d = 2.1 (very large); *p* < 0.001) and 4(95% CI_d_ = −5;−1; d = 1.6 (large); *p* < 0.001 and for week 2 than 3 (95% CI_d_ = −5;−2; d = 1.2 (moderate); *p* < 0.001). 

For the strain values, there was only a significant training effect (F_3,88_ = 16.4; *p* < 0.001) with lower values for week 1 than weeks 3 (95% CI_d_ = −3656; −1547; d = 1.8 (large); *p* < 0.001) and 4 (95% CI_d_ = −2541; −431; d = 1.3 (large); *p* = 0.002) and for week 2 than week 3 (95% CI_d_ = −2973; −863; d = 2.3 (very large); *p* < 0.001) and higher values recorded during week 3 compared to week 4 (95% CI_d_ = 61; 2170; d = −0.6 (small); *p* = 0.03).

For PRS values (Table 2), there was a training effect (F_7,176_ = 8.2; *p* < 0.001) with lower values (i) for session 1 compared to sessions 6 (95% CI_d_ = −2.5; −0.4; d = 0.9 (moderate); *p* = 0.001) and 7 (95% CI_d_ = −2.9; −0.7; d = 1.3 (large); *p* < 0.001), (ii) for session 2 compared to sessions 6 (95% CI_d_ = −3656; −1547; d = 0.9 (moderate); *p* = 0.002) and 7 (95% CI_d_ = −2541; −431; d = 1.4 (large); *p* < 0.001) and (iii) for session 5 in comparison to sessions 6 (95% CI_d_ = −2.5; −0.3; d = 1.0 (moderate); *p* = 0.002) and 7 (95% CI_d_ = −2.9; −0.7; d = 1.6 (large); *p* < 0.001). There was a significant interaction between training and group (F_3,88_ = 6.1; *p* < 0.001). For the RST group, the post hoc indicated lower values (i) for session 1 compared to sessions 6 (95% CI_d_ = −4.4; −1.3; d = 1.8 (large); *p* < 0.001), 7 (95% CI_d_ = −3.9; −0.8; d = 1.6 (large); *p* < 0.001) and 8 (95% CI_d_ = −3.4; −0.3; d = 1.2 (moderate); *p* = 0.006), (ii) for session 2 compared to sessions 6 (95% CI_d_ = −4.7; −1.6; d = 2.2 (very large); *p* < 0.001), 7 (95% CI_d_ = −4.3; −1.2; d = 2.1 (very large); *p* < 0.001) and 8 (95% CI_d_ = −3.7; −0.6; d = 1.5 (large); *p* <0.001), (iii) for session 3 compared to session 6 (95% CI_d_ = −3.1;−0.04; d = 1.4 (large); *p* = 0.038), (iv) for session 4 compared to sessions 6 (95% CI_d_ = −3.9; −0.9; d = 1.8 (large); *p* < 0.001) and 7 (95% CI_d_ = −3.5; −0.5; d = 1.5 (large); *p* = 0.002) and (v) for session 5 compared to sessions 6 (95% CI_d_ = −4.5; −1.4; d = 2.6 (very large); *p* < 0.001), 7 (95% CI_d_ = −4.1; −1.0; d = 2.5 (very large); *p* < 0.001) and 8 (95% CI_d_ = −3.5; −0.5; d = 1.8 (large); *p* = 0.002).

For VO_2max_ values from performance in the 20 m multistage shuttle run test, there was a significant training effect (F_1,66_ = 11.2; *p* = 0.001) indicating an increase from before to after the training program (95% CI_d_ = 1.3; 5.2; d = 0.6 (moderate); *p* = 0.001). There was a significant group effect (F_2,66_ = 10.2; *p* < 0.001) with VO_2max_ values lower in CG compared to RTT (95% CI_d_ = 2.1; 7.9; d = 1.4 (large); *p* < 0.001) and RST (95% CI_d_= 13; 7.1; d = 1.5 (large); *p* = 0.002). An interaction effect was found (F_2,66_ = 3.8; *p* = 0.028) with higher VO_2max_ values at post- compared to pre-training for RTT (95% CI_d_= 2.2; 8.9; d = 1.2 (large); *p* = 0.001) and RST (95% CI_d_ = 1.3; 7.9; d = 1.1 (moderate); *p* = 0.008). At post-training, VO_2max_ values was lower in CG compared to RTT (95% CI_d_ = 3.9; 12.1; d = 0.7 (moderate); *p* < 0.001) and RST (95% CI_d_ = 2.6; 10.9; d = 0.8 (moderate); *p* < 0.001).

For VO_2max_ values collected from the PSTT, there was a training effect (F_1,66_ = 14.868; *p* < 0.001) with VO_2max_ increasing from before to after training (95% CI_d_ = 1.6; 5.0; d = 0.9 (moderate); *p* < 0.001). There was a group effect (F_2,66_ = 12.5; *p* < 0.001) with lower values in CG compared to RTT (95% CI_d_ = 2.3; 7.5; d = 1.8 (large); *p* < 0.001) and RST (95% CI_d_ = 1.5; 6.7; d = 0.9 (moderate); *p* = 0.001).

For the best distance recorded during the 5 m shuttle run test, there was a group effect (F_2,66_ = 6.8; *p* = 0.441) with the RTT showing higher values in comparison to CG (95% CI_d_ = 6.9; 34; d = −1.1 (moderate); *p* = 0.007). Concerning the total distance covered during the 5 m shuttle run test, there was a group effect (F_2,66_ = 4.7; *p* = 0.012) with the RTT group showing higher values in comparison to CG (95% CI_d_ = 10; 117; d = −0.7 (moderate); *p* = 0.014). Moreover, an interaction effect was found between training and group factors (F_2,66_ = 3.3; *p* = 0.043) with higher values for RTT at post-training in comparison to CG (95% CI_d_ = 42; 193; d = −1.5 (large); *p* = 0.001) (Table 3).

Concerning CMJ test performance (Table 3), there was a group effect (F_2,66_ = 5.5; *p* = 0.006) with lower values for CG compared to the RTT (95% CI_d_ = 0.9; 9.4; d = −1.2 (moderate); *p* = 0.012) and RST (95% CI_d_ = 0.5; 9.0; d = −0.9 (moderate); *p* = 0.024).

For the modified agility T-test (Table 3), there was a training effect (F_1,66_ = 10.5; *p* = 0.002) with performance improvement from before to after training (95% CI_d_ = 0.1; 0.7; d = 1.1 (moderate); *p* = 0.002). Moreover, a group effect was observed (F_2,66_ = 8.6; *p* < 0.001) with higher agility performance for the RTT in comparison to CG (95% CI_d_ = −1.1; −0.3; d = 1.4 (large); *p* < 0.001).

For the TSAT (Table 3), there was a training effect (F_1,66_ = 14.8; *p* < 0.001) with performance improvement from before to after training (95% CI_d_ = 0.2; 0.8; d = 1.3 (large); *p* < 0.001). Moreover, a group effect was observed (F_2,66_ = 6.6; *p* < 0.01) with better agility performance for the RTT in comparison to both RST (95% CI_d_ = −0.9; −0.1; d = 1.2 (moderate); *p* = 0.013) and CG (95% CI_d_ = −0.9; −0.1; d = 1.2 (moderate); *p* = 0.004).

For the number of techniques executed with the non-dominant leg during the 10 s specific exercise, there was a training effect (F_1,66_ = 13.7; *p* < 0.001) with performance improvement from before to after training (95% CI_d_ = −2; −1; d = 0.7 (moderate); *p* < 0.001). Moreover, there was a group effect with lower values recorded for CG in comparison to RTT (95% CI_d_ = 4; 1; d = 1.5 (large); *p* < 0.001) and RST (95% CI_d_ = 1; 3, d = 0.6 (moderate); *p* = 0.012) (Table 3).

For the 10 s specific exercise with the dominant leg, there was a training effect (F_1,66_ = 39.8; *p* < 0.001) with the number of techniques increased from before to after training (95% CI_d_ = −3; −2; d = 1.6 (large); *p* < 0.001). Moreover, there was a training group effect (F_2,66_ = 8.5; *p* = 0.001) with higher values for the RTT compared to CG (95% CI_d_ = 1; 3; d = 0.9 (moderate); *p* < 0.001) (Table 3).

For the 1 min specific exercise, a group effect was found (F_2,66_ = 4.801; *p* = 0.011) for the number of techniques executed by the non-dominant leg with the RTT eliciting higher improvements in comparison to CG (95% CI_d_ = 2; 18; d = 0.7 (moderate); *p* = 0.009). In addition, for delta lactate values recorded during 1 min non-dominant leg, only an interaction effect between training and group was found (F_2,66_ = 9.5; *p* < 0.001) with RTT showing lower values after training in comparison to RST (95% CI_d_ = −10.3; −2.4; d = −1.0 (moderate); *p* = 0.001) and CG (95% CI_d_ = −8.4; −0.6; d = −1.2 (large); *p* = 0.017) (Table 3).

Moreover, for the dominant leg during 1 min exercise, there was a training effect (F_1,66_ = 13.4; *p* = 0.001) with number of techniques improving from before to after training (95% CI_d_ = −11; −3; d = 0.9 (moderate); *p* = 0.001). A group effect was found (F_2,66_ = 21.5; *p* < 0.001) with lower values in CG compared to RTT (95% CI_d_ = 9; 21; d = 1.6 (large); *p* < 0.001) and RST (95% CI_d_ = 6; 18; d = 1.30 (large); *p* < 0.001) (Table 3).

In addition, for delta lactate values recorded during 1 min for the dominant leg, only an interaction effect between training and group was found (F_2,66_ = 9.0; *p* < 0.001) with RTT showing lower values after training in comparison to CG (95% CI_d_ = −8.3; −0.8; d = −1.6 (large); *p* < 0.001) and RST (95% CI_d_ = −9.1; −1.4; d = −0.8 (moderate); *p* = 0.004) (Table 3).

## 4. Discussion

The present study showed that weekly TL was higher for week 1 compared to other weeks. Moreover, monotony and strain values were lower in week 1 in comparison to the third and fourth week. Lower values were recorded for PRS for sessions 1, 2, and 5 in comparison to sessions 6 and 7. Considering aerobic performance, VO_2max_ improved after the training period with higher values for RST and RTT in comparison to CG. The total covered distance in the 5 m shuttle run test following the training period was better in RTT group compared to CG. Moreover, CMJ performance did not improve after training with better values recorded for both training groups compared to the CG. For agility performance, RTT elicited significant improvements for both tests (i.e., Modified agility T-test and TSAT) after the 4-week training period. Furthermore, during the 10 s specific exercise, the number of performed techniques with the dominant leg improved for both RTT and RST groups; however, only RTT elicited improvements for the non-dominant leg. For the 1 min specific exercise, the number of techniques executed with the dominant leg improved for the RST and RTT in comparison to CG. Finally, the delta lactate post-training was lower for RTT in both tests (dominant and non-dominant leg) compared to RST and to CG. Thus, the initial hypothesis of the study was partially confirmed.

Despite the increase in the training volume from week 1 to week 3, the perceived mean TL decreased from the beginning to the last week, which confirms that athletes were able to adapt psychologically to the load of the training program. Nevertheless, monotony and strain were higher in weeks 3 and 4 in comparison to week 1, which corresponds to a high level of fatigue being the result of the cumulative effects of TL [29]. Monotony values were higher than 2, indicating that the training was monotonous [24]. Moreover, high strains during the training program may induce physiological disorders leading to the homeostasis disruption [26]. Monotony and strain did not change accordingly to the two modalities of training, suggesting that these parameters were not affected by exercise mode; but by the training intensity [24]. It is well known that high monotony and strain indicate that there is an unbalance between the training and the recovery process [24]. During the present study, PRS score was 4 (somewhat recovered) for session 1 and approximately 6 (moderately recovered) [25] for sessions 6 and 7, which may confirm that the intense training induced an unbalance for the recovery process despite the PRS score improvement along the period.

It has been indicated that aerobic fitness is a key factor that positively affects taekwondo performance in athletes by improving their recovery process, which is paramount to successfully perform subsequent high-intensity actions [7]. In the present study, VO_2max_ (calculated in the two different aerobic tests) improved significantly following the 4 weeks of training in both RST and RTT. Similar results were reported in other studies with combat sports athletes training HIIT additional to their regular training regime with durations ranging from 4–7 weeks, for taekwondo [7], karate [8], boxing [13], judo [30] and wrestling [4]. However, other studies showed that HIIT was not effective in improving VO_2max_ in adult athletes when added to regular judo training program [6,31]. The present study was the first to use both non-specific and taekwondo-specific aerobic power tests to analyze the effects of taekwondo-specific repeated high-intensity technique training and repeated sprint running. Based on the results of the present study, it seems that both non-specific and taekwondo-specific high intensive interval training can induce aerobic power improvements and that both non-specific and taekwondo-specific tests can be used to detect such changes. Therefore, for aerobic fitness improvement purposes, both specific and non-specific highly intensive interval training and tests can be valuable tools during the HIIT prescription and evaluation in adolescent taekwondo athletes.

Anaerobic performance in the 5 m shuttle run test, (total distance and best distance) did not change with training. This finding is not in line with similar studies, demonstrating that the same training program elicited higher mean and peak power output recorded during the 30 s Wingate anaerobic test among wrestlers with significant differences compared to CG [4]. Moreover, Monks et al. [7] reported that 11 sessions of HIIT over 4 weeks improved anaerobic performance during a 30 s Wingate test. This difference may be explained by the fact that while athletes in the present study exclusively trained RST and normal taekwondo training, whereas in the study of Farzader et al. [4], athletes performed RST and additional physical conditioning training such as weight training and plyometrics which may influenced positively anaerobic fitness. Moreover, differences may also be attributed to the tests used (Wingate anaerobic test vs. 5 m shuttle run test) [4,7] as well as the frequency of training sessions (3 sessions/week [7] vs. 2 sessions/week in the present study).

The absence of neuromuscular performance improvement (i.e., CMJ in the present study) is not in agreement with the study of Monks et al. [7], which reported that 11 sessions of HIIT based on running at 85~100% of maximum HR over 4 weeks improved vertical jump performance. Nevertheless, Buchheit et al. [32] reported that 10 weeks of HIIT among adolescent athletes resulted in trivial changes in CMJ performance. In the same consideration, a recent meta-analysis showed that HIIT had generally a small effect on jumping height in young and adolescent athletes [14]. Although both running and specific repeated high-intensity taekwondo techniques were used as two modalities of training during the present study, the program was not sufficient to induce vertical jump performance improvements. This difference may be attributed to the difference between volume and frequency of training, the lack of overload and focus on power training [33]. Moreover, this finding confirms that training based on short sprints did not yield improvements in jump ability and that technical aspects of jump performance may need to be practiced during HIIT if meaningful improvements in performance are to be conferred [33]. Furthermore, taekwondo athletes already perform many jumps and powerful actions during their habitual training routine, which leads to the assumption that the addition of HIIT was not sufficient to contribute to further improvements.

Agility performance was improved for RTT after the training period. Similar results were reported by Monks et al. [7] who revealed improvements in agility (T-test) after 11 sessions of running-based HIIT. Moreover, Fernandez-Fernandez et al. [34] reported a meaningful, but not statistically significant, change in the 505-agility test after combining high-intensity training and tennis specific drill training. The present study showed improvement of the modified T-test and TSAT, with moderate to large meaningful improvements, respectively. This large improvement for the TSAT can be explained by the fact that technical execution was properly and highly improved after repeated technique training, positively influencing the athlete’s performance during this test, which is composed by a combination between side-steps with techniques actions. Finally, for power-related variables, the training program did not induce improvements in CMJ performance with better agility performance which confirms that jump-specific measures of explosive strength may not always translate to a holistic enhancement of power-related performance and vice versa [33].

During the 10 s and 1 min specific exercises, the number of techniques for the non-dominant and dominant legs improved largely after the training period mainly for RTT, indicating an important anaerobic development. This improvement can be explained by the fact that the technique repetition through the training program, which combined both severe fatigue and technical actions, induced technique adaptations [35]. These adaptations could be mainly based on strength improvements in specific muscles activated during the taekwondo technique performed (i.e., mainly biceps femoris and knee extensor muscles) [35].

Finally, RTT yielded practically significant lower delta blood lactate values following the training period. This improvement supports the effectiveness of this modality of training to induce adaptations such as an increasing oxidative adenosine triphosphate (ATP) synthesis, which decrease the contribution of anaerobic ATP resynthesis and slows down the blood lactate accumulation [36]. Despite the lack of improvement in delta lactate following the 5 m shuttle run test, the decrease found during the 1 min specific exercise supports the idea that assessment of training effects in taekwondo athletes should be taekwondo-specific.

We acknowledge limitations in the present study. In fact, while we assessed the different components of fitness of taekwondo athletes with generic and specific valid and reliable tests, the study did not establish whether training induced changes in such parameters translate to meaningful improvements in sport performance under competitive conditions such as reporting time–motion and technical and tactical performance from combat simulations. Moreover, the present study analyzed adolescent taekwondo athletes and, therefore, our results may not apply to adult taekwondo athletes.

## 5. Conclusions

The present study showed that repeated sprint and repeated technique training, performed with high intensities, were two effective training modalities to induce aerobic improvements. Moreover, RTT induced improvements in agility and the total number of techniques performed during the specific exercises with lower delta blood lactate at post-training compared to RST group. The importance of this program is that its temporal structure mimics that of the taekwondo competition, using the specific technique mostly used in combats and would likely induce specific adaptations beneficial for successful performance in competition.

These findings suggest the effectiveness of a low-volume high-intensity training based on repetitions of taekwondo-specific techniques added to regular training regime in inducing specific improvements toward taekwondo activity. Moreover, increased monotony and strain through the training period should encourage coaches to pay attention to the recovery–stress state of young athletes when applying these training modalities, especially in the lead-up to competition, in order to avoid non-functional overreaching.

## Figures and Tables

**Table 1:** Weekly training load, monotony and strain during the training intervention (values are mean ± SD) for RTT (*n* = 12), RST (*n* = 12) and CG (*n* = 12).

Week	Group	Training Load (a.u.)	Monotony (a.u.)	Strain (a.u.)
Week 1	RTT	662 ± 26	3.6 ± 0.8	2431 ± 586
	RST	680 ± 64	3.7 ± 1.1	2512 ± 618
	CG	671 ± 49 ^a,b,c^	3.7 ± 0.9 ^d^	2471 ± 603 ^d^
Week 2	RTT	633 ± 40	4.4 ± 0.9	2752 ± 665
	RST	588 ± 73	6.1 ± 1.4	3558 ± 801
	CG	610 ± 62	5.2 ± 1.5	3155 ± 841
Week 3	RTT	617 ± 83	8.3 ± 3.1	5207 ± 2187
	RST	580 ± 67	8.4 ± 2.8	4940 ± 1703
	CG	604 ± 77	8.4 ± 2.9	5074 ± 1975
Week 4	RTT	585 ± 76	6.1 ± 2.1	3669 ± 1543
	RST	580 ± 59	7.4 ± 2.6	4247 ± 1437
	CG	583 ± 68	6.8 ± 2.4	3958 ± 1522

a.u. = arbitrary units; ^a^ different from week 2 (*p* = 0.009); ^b^ different from week 3 (*p* = 0.003); ^c^ different from week 4 (*p* < 0.001); ^d^ different from week 3 and 4 (*p* < 0.001); RTT: repeated technique training group; RST: repeated sprint training group; CG: control group.

**Table 2:** Perceived recovery status scores recorded during the different training sessions (values are mean ± SD) for the RTT (*n* = 12), RST (*n* = 12) and CG (*n* = 12).

Group		Session 1	Session 2	Session 3	Session 4	Session 5	Session 6	Session 7	Session 8
PRS (a.u)	RTT	5 ± 1	5 ± 1	5 ± 1	5 ± 1	5 ± 1	4.5 ± 1	6 ± 1	5 ± 1
	RST	4 ± 2 ^d,e^	4 ± 2 ^f^	6 ± 1 ^g^	5 ± 2 ^d,h^	4 ± 1 ^d,i^	7 ± 1	7 ± 1	6 ± 1
	CG	4 ± 2 ^a,b^	4 ± 1 ^b,c^	5 ± 1	5 ± 1	4 ± 1 ^b,c^	6 ± 2	6 ± 1	5 ± 1

a.u. = arbitrary units; ^a^ different from session 6 (*p* = 0.001); ^b^ different from session 7 (*p* < 0.001); ^c^ different from session 6 (*p* = 0.002); ^d^ different from session 6 and 7 (*p* < 0.001); ^e^ different from session 8 (*p* = 0.006); ^f^ different from session 6, 7 and 8 (*p* < 0.001); ^g^ different from session 6 (*p* = 0.038); ^h^ different from session 7 (*p* = 0.002); ^i^ different from session 8 (*p* = 0.002); RTT: repeated technique training group; RST: repeated sprint training group; CG: control group.

**Table 3:** Physiological and performance parameters before and after the training period (values are mean ± SD) for the repeated technique training group (RTT; *n* = 12), the repeated sprint training group (RST; *n* = 12) and the control group (CG; *n* = 12).

Tests	Performance	RTT		RST		CG		Overall	
		Before Training	After Training	Before Training	After Training	Before Training	After Training	Before Training	After Training
20 m multistage shuttle run test	VO_2max_ (mL/kg/min)	39.8 ± 3.1 ^b^	45.4 ± 4.2 ^a, b^	39.6 ± 3.6 ^b^	44.2 ± 3.9 ^a,b^	37.2 ± 4.9	36.8 ± 5.4	38.8 ± 4.0	42.0 ± 5.9
PSTT		51.1 ± 4.9 ^b^	56.4 ± 3.7 ^a,b^	50.9 ± 3.4 ^b^	55.0 ± 2.9 ^a,b^	48.3± 3.2	49.5 ± 3.5	50.1 ± 4.0	53.5 ± 4.5
5 m shuttle run test	Best distance (m)	134 ± 26 ^b^	140 ± 19 ^b^	126 ± 22	129 ± 16	118 ± 18	115 ± 12	126 ± 8	128 ± 12
	Fatigue index (%)	81 ± 14	82 ± 5	85 ± 9	85 ± 5	86 ± 8	80 ± 12	84 ± 3	82 ± 3
	Total distance covered (m)	678 ± 85 ^b^	741 ± 72 ^b^	642 ± 40	678 ± 59	668 ± 98	624 ± 84	663 ± 19	681 ± 59
	Δ[La] (mmol/L)	15.0 ± 4.4	10.4 ± 3.0	13.0 ± 3.6	13.5 ± 3.7	14.5 ± 4.2	14.1 ± 4.4	14.2 ± 4.1	12.6 ± 4.0
Countermovement jump	Jump height (cm)	29.3 ± 7.2 ^a^	31.4 ± 5.9 ^a^	29.4 ± 6.4 ^a^	30.5 ± 6.8 ^a^	25.1 ± 4.7	25.3 ± 4.7	27.9 ± 2.5	27.9 ± 3.7
Agility	TAST (s)	6.72 ± 0.5 ^a,c^	6.1 ± 0.3 ^a,b,c^	7.08 ± 0.7	6.7 ± 0.7 ^b^	7.03 ± 0.4	6.9 ± 0.4 ^b,c^	6.94 ± 0.2	6.6 ± 0.4
	T-test (s)	6.5 ± 0.5 ^a^	6.0 ± 0.5 ^a,b^	6.8 ± 0.6	6.3 ± 0.6 ^b^	7.18 ± 0.7	6.8 ± 0.6 ^b^	6.82 ± 0.3	6.4 ± 0.4
1 min repeated technique exercise	DL (tech/min)	101 ± 10	113 ± 10 ^a,b^	101 ± 6	108 ± 9 ^a,b^	90 ± 9	93 ± 7 ^a^	97 ± 6	105 ± 10
	Δ[La]DL (mmol/L)	12.8 ± 4.9	8.8 ± 3.4 ^b,c^	9.2 ± 3.1	11.3 ± 4.1 ^b^	7.7 ± 3.3	13.3 ± 4.0	9.9 ± 4.3	11.1 ± 4.2
	NDL (tech/min)	99 ± 13 ^b^	108 ± 14 ^b^	96 ± 7	99 ± 14	91 ± 10	95 ± 9	95 ± 4	100 ± 7
	Δ[La]NDL (mmol/L)	13.8 ± 5.3	11.1 ± 3.7	10.3 ± 2.5	14.7 ± 4.6	9.5 ± 3.9	14.0 ± 4.2	11.2 ± 4.4	13.3 ± 4.4
10 s repeated technique exercise	DL (tech/s)	22 ± 1	26 ± 2 ^a,b^	22 ± 2	24 ± 2 ^a^	21 ± 2	21 ± 2	22 ± 1	24 ± 2
	NDL (tech/s)	23 ± 2 ^b^	25 ± 2 ^a,b^	21 ± 2	24 ± 2 ^a,b^	20 ± 1	21 ± 1	21 ± 1	23 ± 2

^a^ different from before training; ^b^ different from CG; ^c^ different from RST.

## References

[1] Bridge C.A., Ferreira da Silva Santos J., Chaabène H., Pieter W., Franchini E. (2014). Physical and physiological profiles of taekwondo athletes. Sports Med..

[2] World Taekwondo Website WT Competition Rules & Interpretation. http://www.worldtaekwondo.org/rules/.

[3] Bridge C.A., Jones M.A., Drust B. (2011). The activity profile in international Taekwondo competition is modulated by weight category. Int. J. Sports Physiol. Perform..

[4] Farzad B., Gharakhanlou R., Agha-Alinejad H., Curby D.G., Bayati M., Bahraminejad M., Mäestu J. (2011). Physiological and performance changes from the addition of a sprint interval program to wrestling training. J. Strength Cond. Res..

[5] Haddad M., Chaouachi A., Wong D.P., Castagna C., Chamari K. (2011). Heart rate responses and training load during nonspecific and specific aerobic training in adolescent taekwondo athletes. J. Hum. Kinet..

[6] Kim J., Lee N., Trilk J., Kim E.J., Kim S.Y., Lee M., Cho H.C. (2011). Effects of sprint interval training on elite Judoists. Int. J. Sports Med..

[7] Monks L., Seo M.W., Kim H.B., Jung H.C., Song J.K. (2017). High-intensity interval training and athletic performance in Taekwondo athletes. J. Sports Med. Phys. Fit..

[8] Ravier G., Dugué B., Grappe F., Rouillon J.D. (2009). Impressive anaerobic adaptations in elite karate athletes due to few intensive intermittent sessions added to regular karate training. Scand. J. Med. Sci. Sports.

[9] Vasconcelos B.B., Protzen G.V., Galliano L.M., Kirk C., Del Vecchio F.B. (2020). Effects of High-Intensity Interval Training in Combat Sports: A Systematic Review with Meta-Analysis. J. Strength Cond. Res..

[10] Buchheit M., Laursen P.B. (2013). High-intensity interval training, solutions to the programming puzzle. Part II: Anaerobic energy, neuromuscular load and practical applications. Sports Med..

[11] Franchini E., Cormack S., Takito M.Y. (2019). Effects of High-Intensity Interval Training on Olympic Combat Sports Athletes’ Performance and Physiological Adaptation: A Systematic Review. J. Strength Cond. Res..

[12] Seo M.W., Lee J.M., Jung H.C., Jung S.W., Song J.K. (2019). Effects of Various Work-to-rest Ratios during High-intensity Interval Training on Athletic Performance in Adolescents. Int. J. Sports Med..

[13] Kamandulis S., Bruzas V., Mockus P., Stasiulis A., Snieckus A., Venckunas T. (2018). Sport-Specific Repeated Sprint Training Improves Punching Ability and Upper-Body Aerobic Power in Experienced Amateur Boxers. J. Strength Cond. Res..

[14] Engel F.A., Ackermann A., Chtourou H., Sperlich B. (2018). High-Intensity Interval Training Performed by Young Athletes: A Systematic Review and Meta-Analysis. Front. Physiol..

[15] Chtourou H., Souissi N. (2012). The effect of training at a specific time of day: A review. J. Strength Cond. Res..

[16] Léger L.A., Mercier D., Gadoury C., Lambert J. (1988). The multistage 20 meter shuttle run test for aerobic fitness. J. Sports Sci..

[17] SantAna J., Franchini E., Murias J.M., Diefenthaeler F. (2019). Validity of a Taekwondo-Specific Test to Measure VO2peak and the Heart Rate Deflection Point. J. Strength Cond. Res..

[18] Cazorla G., Boussaidi L., Godemet M. (2004). Evaluation du rugbyman sur le terrain. Actes du Congrès Médical de la Fédération Française de Rugby: Pathologies du Rugbyman, Epaule, Genoux, Rachis, Physiologie.

[19] Sassi R.H., Dardouri W., Yahmed M.H., Gmada N., Mahfoudhi M.E., Gharbi Z. (2009). Relative and absolute reliability of a modified agility T-test and its relationship with vertical jump and straight sprint. J. Strength Cond. Res..

[20] Chaabene H., Negra Y., Capranica L., Bouguezzi R., Hachana Y., Rouahi M.A., Mkaouer B. (2018). Validity and Reliability of a New Test of Planned Agility in Elite Taekwondo Athletes. J. Strength Cond. Res..

[21] Bouhlel E., Jouini A., Gmada N., Nefzi A., Ben Abdallah K., Tabka Z. (2006). Heart rate and blood lactate responses during Taekwondo training and competition. Sci. Sports.

[22] World Medical Association (2013). Declaration of Helsinki: Ethical principles for medical research involving human subjects. JAMA.

[23] Foster C., Florhaug J.A., Franklin J., Gottschall L., Hrovatin L.A., Parker S., Doleshal P., Dodge C. (2001). A new approach to monitoring exercise training. J. Strength Cond. Res..

[24] Foster C. (1998). Monitoring training in athletes with reference to overtraining syndrome. Med. Sci. Sports Exerc..

[25] Laurent C.M., Green J.M., Bishop P.A., Sjökvist J., Schumacker R.E., Richardson M.T., Curtner-Smith M. (2011). A practical approach to monitoring recovery: Development of a perceived recovery status scale. J. Strength Cond. Res..

[26] Halson S.L. (2014). Monitoring training load to understand fatigue in athletes. Sports Med..

[27] Rocha F.P.S., Louro H., Matias R., Brito J., Costa A.M. (2016). Determination of Aerobic Power Through a Specific Test for Taekwondo -A Predictive Equation Model. J. Hum. Kinet..

[28] Hopkins W.G. A New View of Statistics: Effect Magnitudes. https://www.sportsci.org/resource/stats/effectmag.html.

[29] Freitas V.H., Nakamura F.Y., Miloski B., Samulski D., Bara-Filho M.G. (2014). Sensitivity of physiological and psychological markers to training load intensification in volleyball players. J. Sports Sci. Med..

[30] Lee N., Kim J., Hyung G.A., Park J.H., Kim S.J., Kim H.B., Jung H.S. (2015). Training Effects on Immune Function in Judoists. Asian J. Sports Med..

[31] Franchini E., Julio U.F., Panissa V.L.G., Lira F.S., Gerosa-Neto J., Branco B.H. (2016). High-Intensity Intermittent Training Positively Affects Aerobic and Anaerobic Performance in Judo Athletes Independently of Exercise Mode. Front. Physiol..

[32] Buchheit M., Laursen P.B., Kuhnle J., Ruch D., Renaud C., Ahmaidi S. (2009). Game-based training in young elite handball players. Int. J. Sports Med..

[33] Tolfrey K., Smallcombe J., Armstrong N., van Mechelen W. (2017). High-intensity interval training. Textbook of Children’s Sport and Exercise Medicine.

[34] Fernandez-Fernandez J., Sanz D., Sarabia J.M., Moya M. (2017). The Effects of Sport-Specific Drills Training or High-Intensity Interval Training in Young Tennis Players. Int. J. Sports Physiol. Perform..

[35] Quinzi F., Camomilla V., Di Mario A., Felici F., Sbriccoli P. (2016). Repeated Kicking Actions in Karate: Effect on Technical Execution in Elite Practitioners. Int. J. Sports Physiol. Perform..

[36] Olek R.A., Kujach S., Ziemann E., Ziolkowski W., Waz P., Laskowski R. (2018). Adaptive Changes after 2 Weeks of 10-s Sprint Interval Training with Various Recovery Times. Front. Physiol..

